# Supplementary material for: Functional annotations of three domestic animal genomes provide vital resources for comparative and agricultural research
Source: Nat Commun. 2021 Mar 23;12:1821. doi: 10.1038/s41467-021-22100-8 (PMC7988148; doi:10.1038/s41467-021-22100-8)
Supplement: Supplementary file 1 — Supplementary Information [file 41467_2021_22100_MOESM1_ESM.pdf]

Functional annotations of three domestic animal genomes provide vital resources for comparative and agricultural research

Supplementary Table 1. **Depth of sequencing and quality of data.** Sequencing and quality metrics data for five ChIP-seq marks (H3K4me3, H3K4me1, H3K27ac, H3K27me3, CTCF) and chromatin accessibility assay (DNase-seq on chicken samples, ATAC-seq on pig and cattle samples). See Methods for definitions and descriptions of the quality metrics used.

	Chicken	Pig	Cattle
Total ChIP-seq reads generated	5,021,232,911	4,281,659,559	6,813,035,002
Chromatin accessibility reads	805,274,643	1,038,779,370	1,190,252,653
Average ChIP-seq reads per sample	62,765,411	53,520,744	85,162,938
Average chromatin accessibility reads per sample	67,500,862	64,923,711	79,350,177
Average NRF	0.73 ± 0.14	0.78 ± 0.07	0.66 ± 0.17
Average PBC1	0.74 ± 0.15	0.79 ± 0.07	0.68 ± 0.15
Average PBC2	5.18 ± 2.55	5.29 ± 1.71	4.09 ± 1.67
Average NSC	1.18 ± 0.20	1.28 ± 0.32	1.34 ± 0.41
Average RSC	2.24 ± 1.97	1.39 ± 0.41	2.59 ± 1.86
Average JSD	0.22 ± 0.14	0.24 ± 0.13	0.16 ± 0.13
Average FRiP	0.31 ± 0.16	0.28 ± 0.14	0.18 ± 0.13

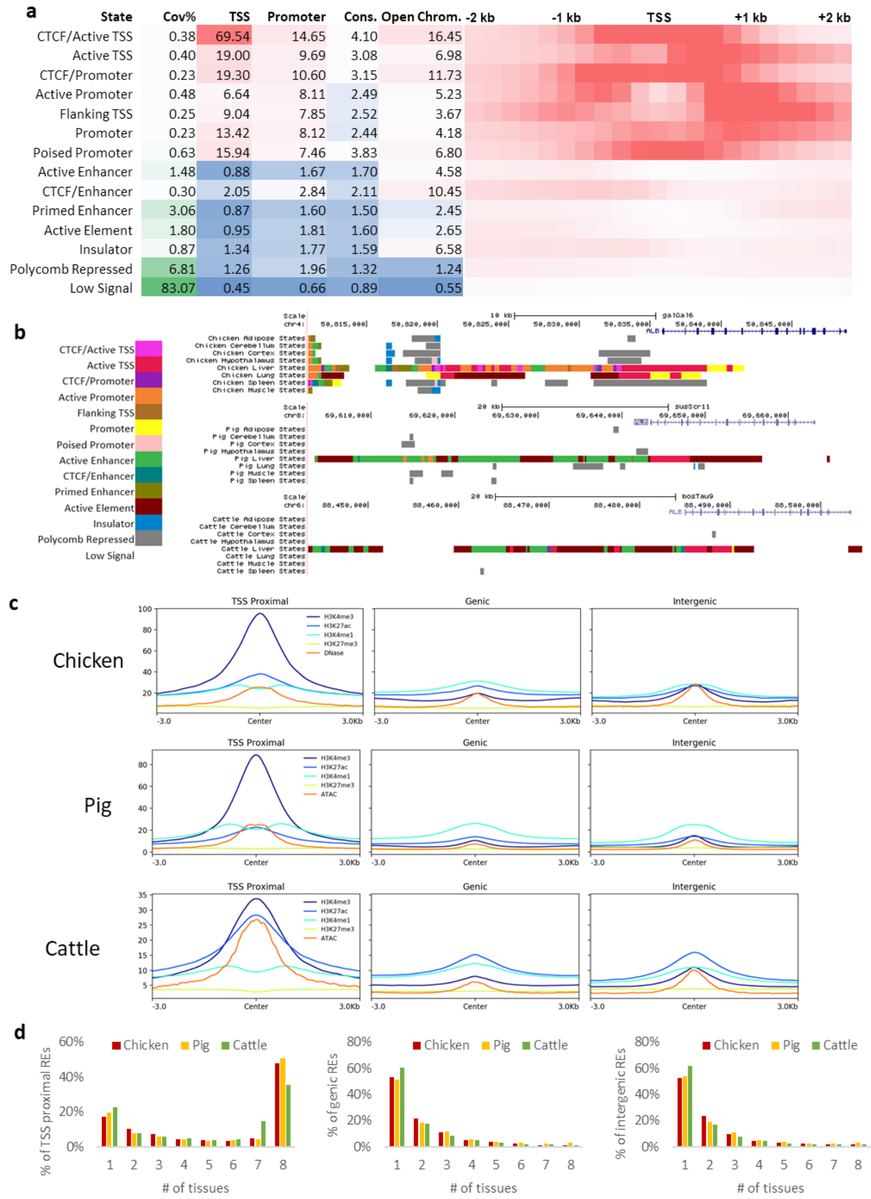

Supplementary Figure 1. **Active regulatory regions**. **a** For each state, averaged across species and tissues, the genome coverage, enrichment at TSS, promoter regions (defined as 2 kb upstream of TSS), conserved elements, open chromatin regions, and the enrichment in 4 kb windows centered on each annotated TSS (right). **b** Chromatin state predictions at the albumin (ALB) locus, which is highly expressed in liver. **c** Average enrichment of histone modifications and chromatin accessibility in regulatory elements. **d** The percentage of regulatory elements active in different numbers of tissues.

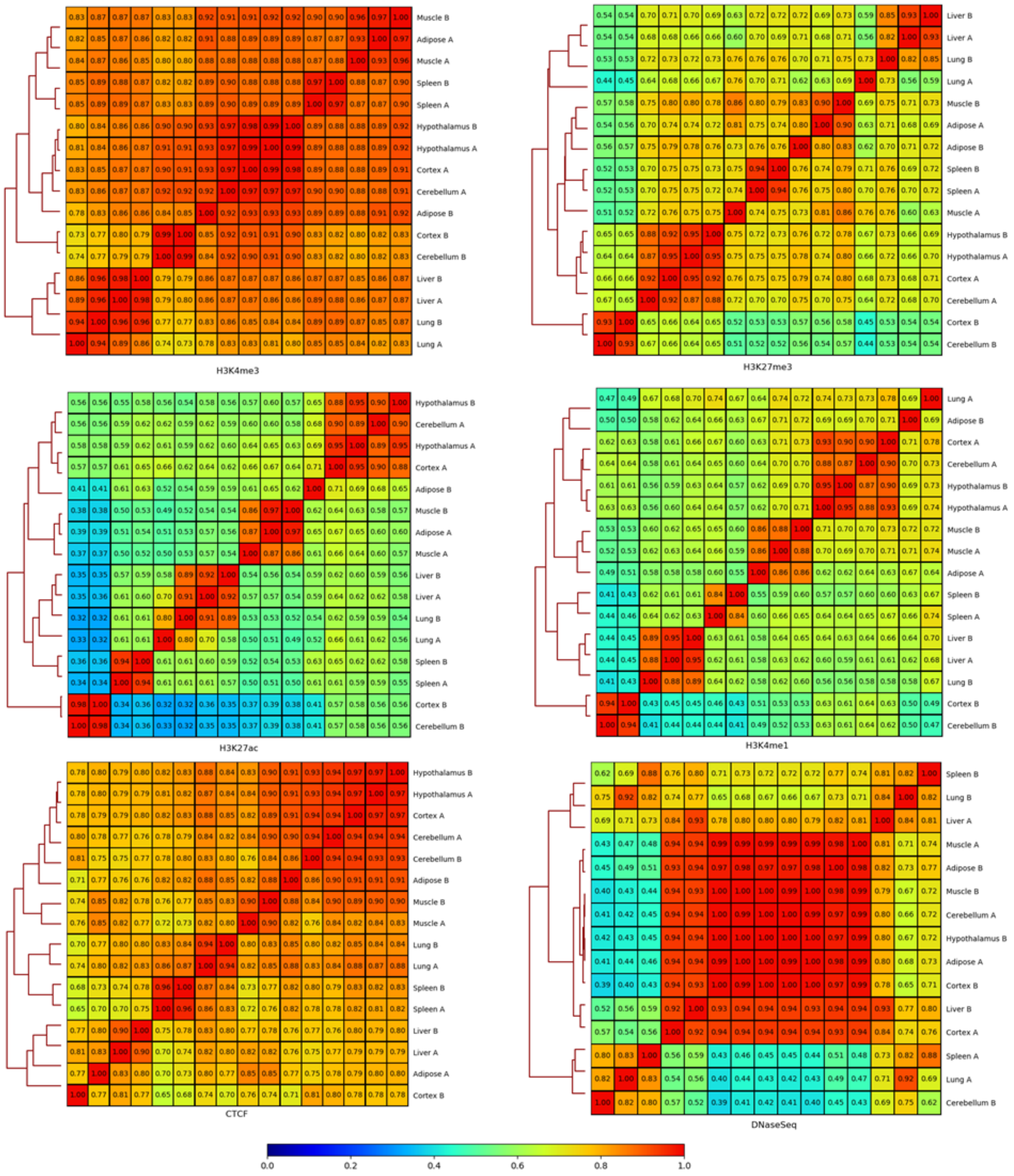

Supplementary Figure 2. **Hierarchical clustering of chicken data.** Hierarchical clustering of the five ChIP-seq marks and DNase-seq based on the Pearson correlation of read depth distribution across the genome.

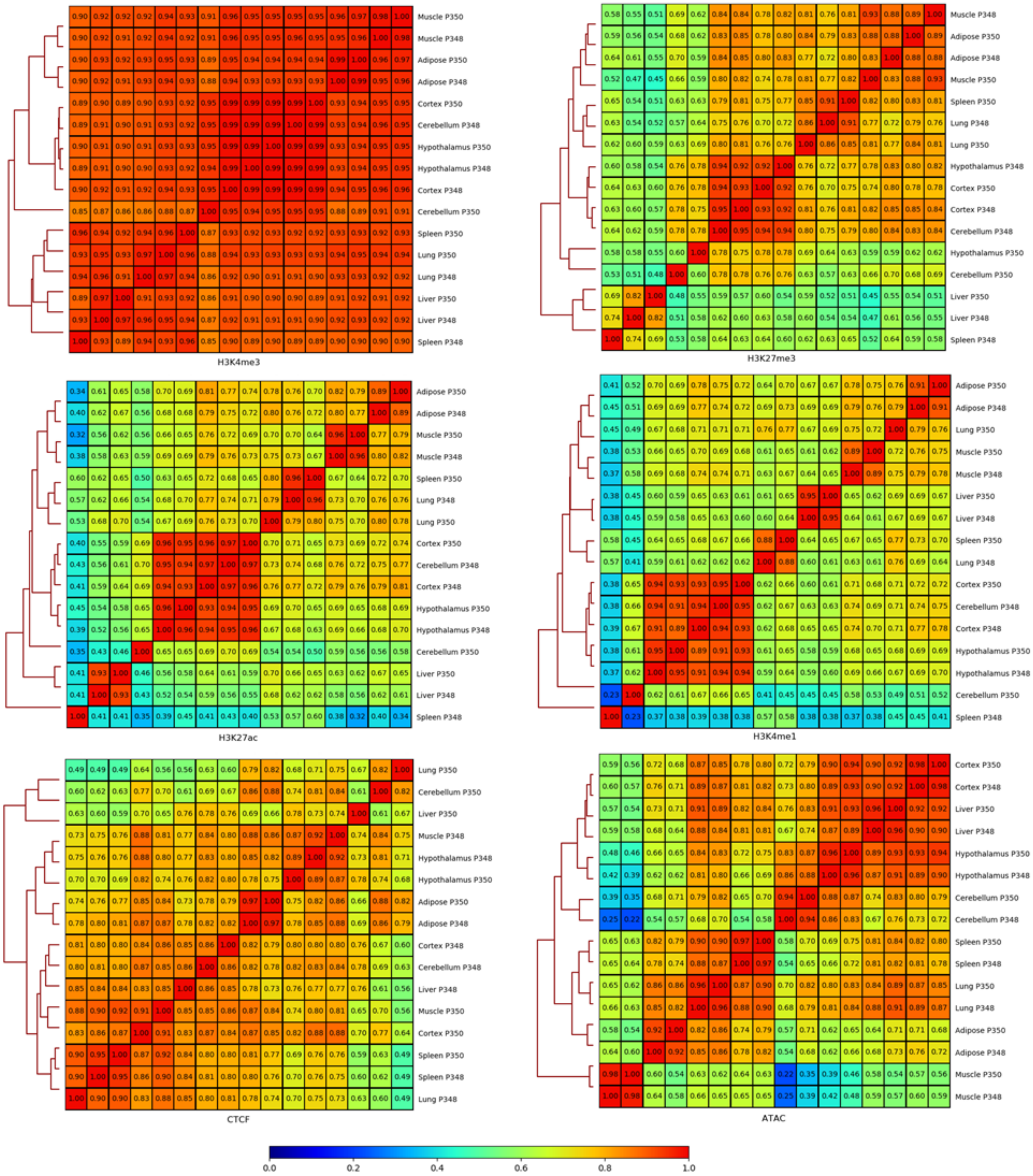

Supplementary Figure 3. **Hierarchical clustering of pig data.** Hierarchical clustering of the five ChIP-seq marks and ATAC-seq based on the Pearson correlation of read depth distribution across the genome.

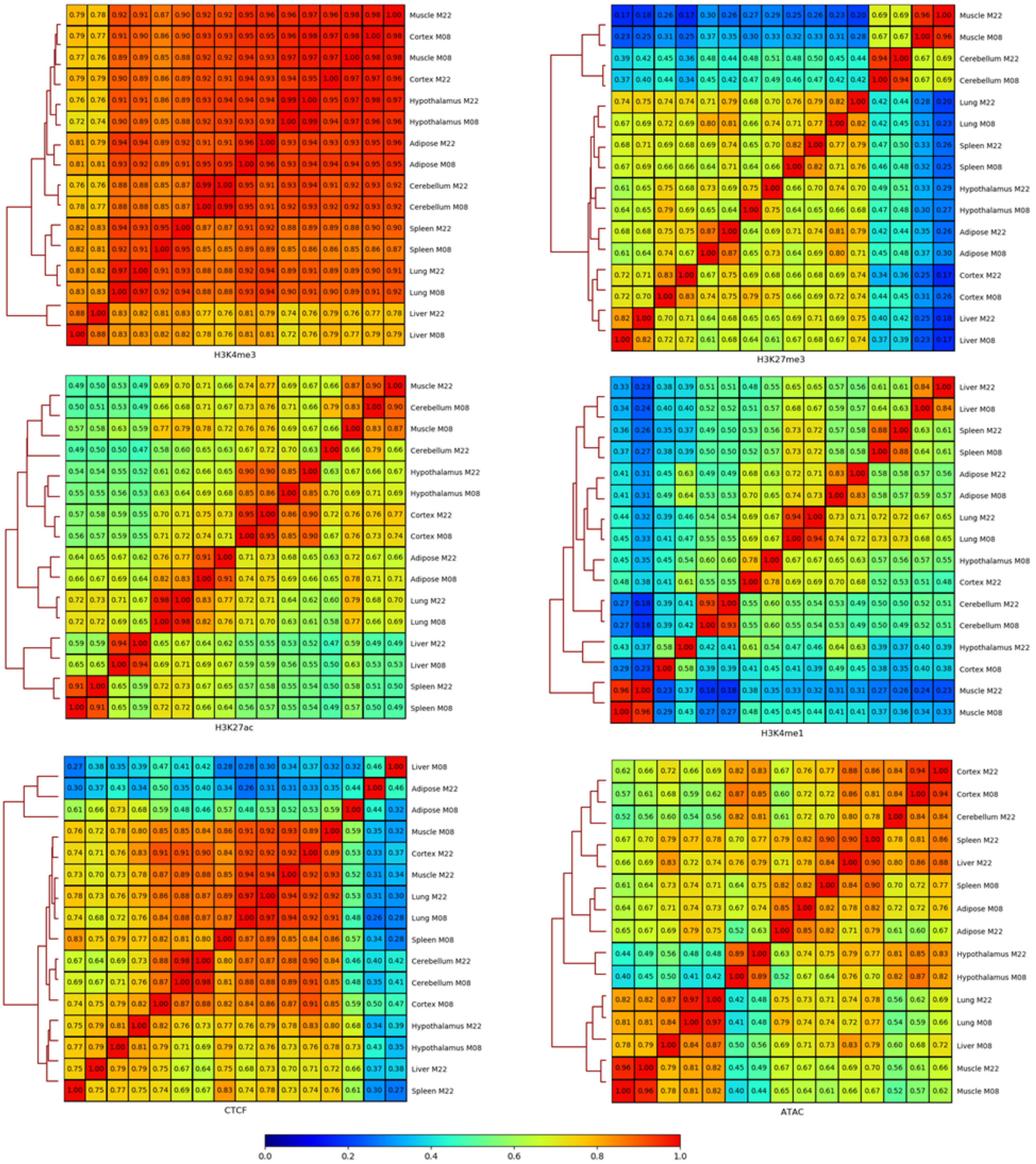

Supplementary Figure 4. **Hierarchical clustering of cattle data.** Hierarchical clustering of the five ChIP-seq marks and ATAC-seq based on the Pearson correlation of read depth distribution across the genome.

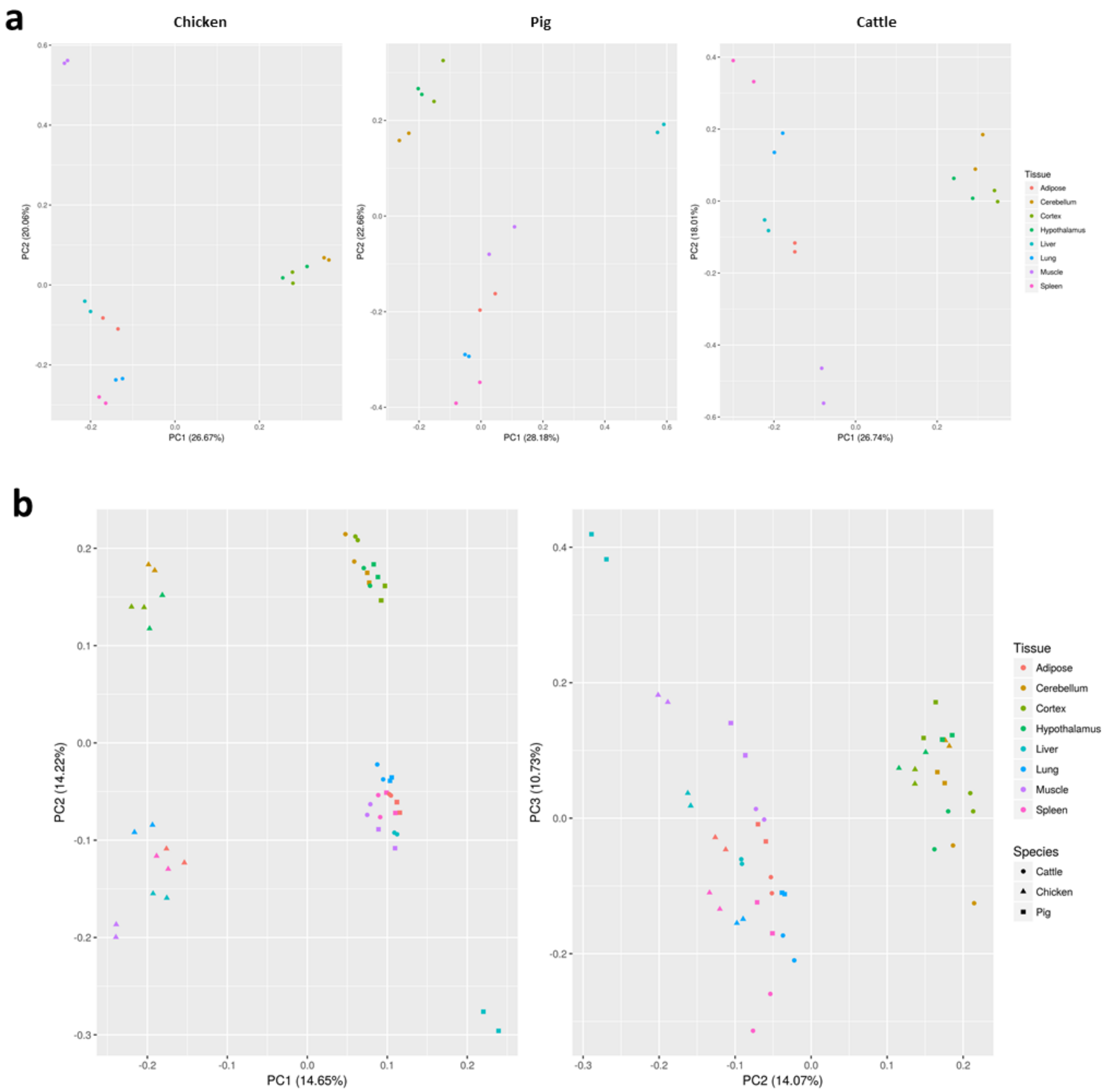

Supplementary Figure 5. **Principal component analysis of gene expression. a** Principal component plot of TMM-normalized RNA-seq counts of all annotated genes in chickens (left), pigs (middle), and cattle (right). **b** Principal component plot of TMM-normalized RNA-seq counts of all one-to-one orthologs across the three species, plotting PC1 vs. PC2 (left) and PC2 vs. PC3 (right).

a

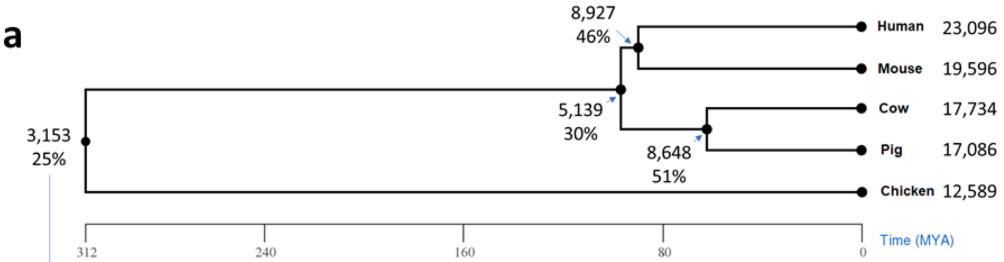

Tissue	Active promoters	Tissue-specific activity	Genes with conserved tissue-specific promoters
Adipose	2,793	20	CREB5, SGK1, SVEP1, +16 more
Brain	2,860	50	BRINP1, SNAP91, SYNGR3, +42 more
Liver	2,978	144	ALB, HAL, NRK, +136 more
Lung	2,718	6	COL6A1, CRISPLD1, DNAJA4, +2 more
Spleen	2,691	7	IRF4, IKZF3, SCIN, +4 more

b

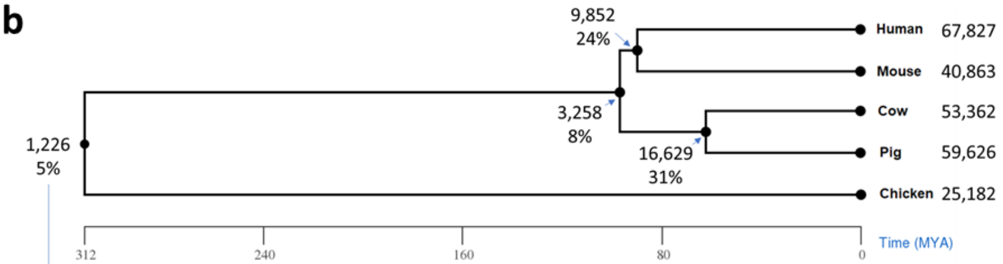

Tissue	Active genic enhancers	Tissue-specific activity	Predicted target genes in pigs
Adipose	713	33	ADIP, MXI1, CACHD1, PXDN, +2 more
Brain	880	118	GRID2, SNCA, SCAI, +27 more
Liver	695	55	CNMD, TRAP1, FN1, +19 more
Lung	787	29	TRAM2, MAP7D2
Spleen	755	32	SOX2, MAP6D1, CHEK2, TCP11

c

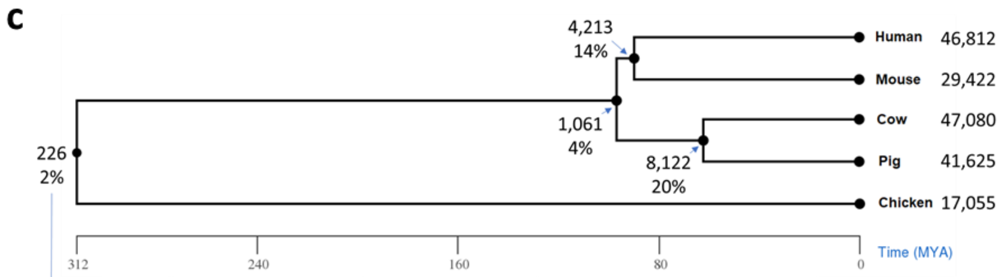

Tissue	Active intergenic enhancers	Tissue-specific activity	Predicted target genes in pigs
Adipose	134	11	C14orf39, SIX1, SIX4, TMEM30B
Brain	142	17	RORB, SERP2
Liver	133	18	PPP1R3C, HECTD2
Lung	145	3	-
Spleen	146	2	-

Supplementary Figure 6. **Conserved regulatory elements in each lineage.** Conservation of (**a**) TSS proximal, (**b**) genic, and (**c**) intergenic regulatory elements within each phylogenetic lineage. For elements conserved across all species, the number active in each tissue, active only in one tissue, and the genes predicted as regulatory targets of those active in only a single tissue are shown.

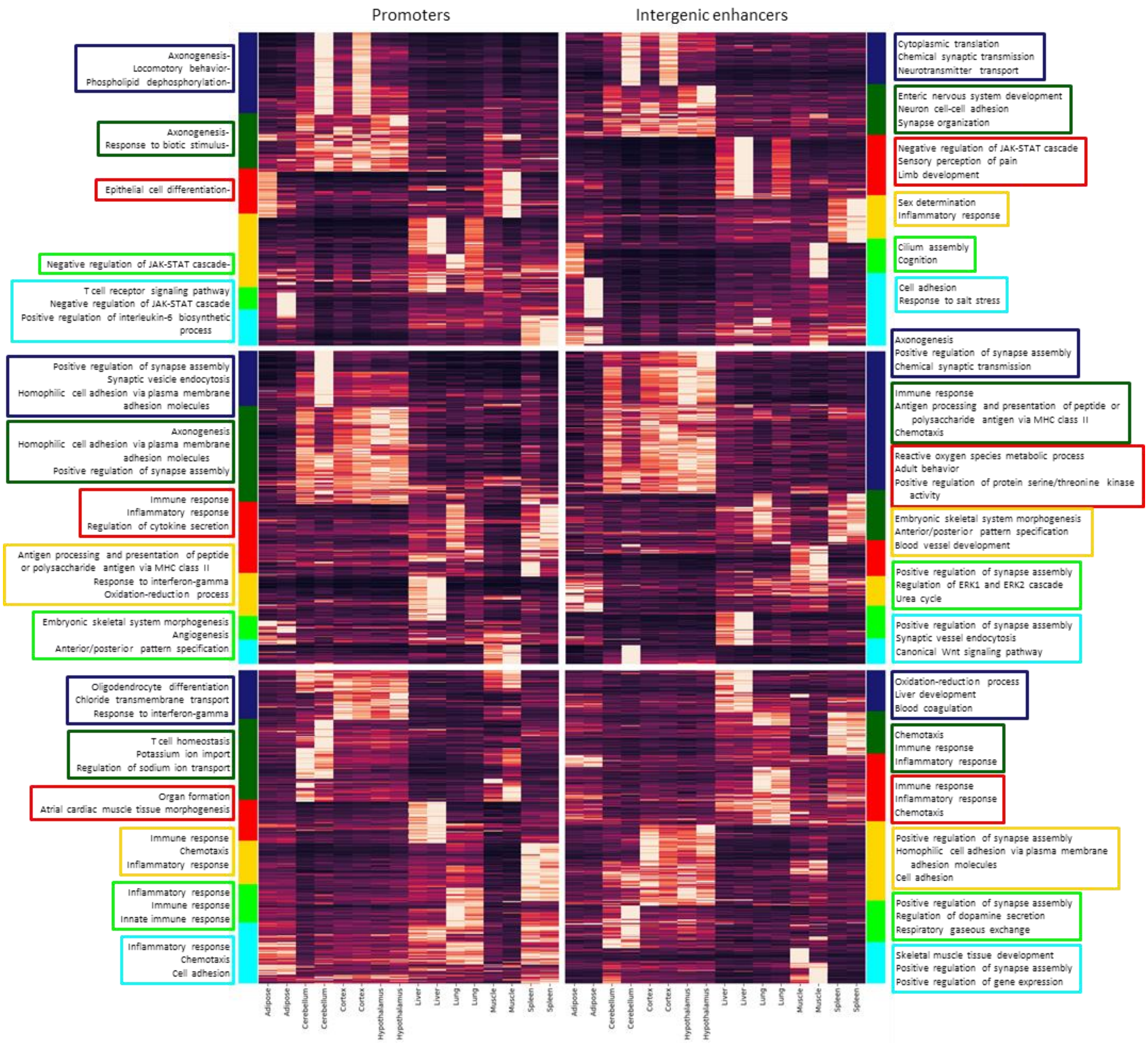

Supplementary Figure 7. **Clustering of regulatory elements and GO term enrichment of predicted targets.** Clustering of all REs with a predicted target gene based on H3K27ac signal, and top 3 GO terms enriched in genes predicted as targets of each cluster. Heatmaps are organized with species as rows (top: chicken, middle: pig, bottom: cattle) and columns as the group of RE (left: promoters, right: intergenic enhancers).

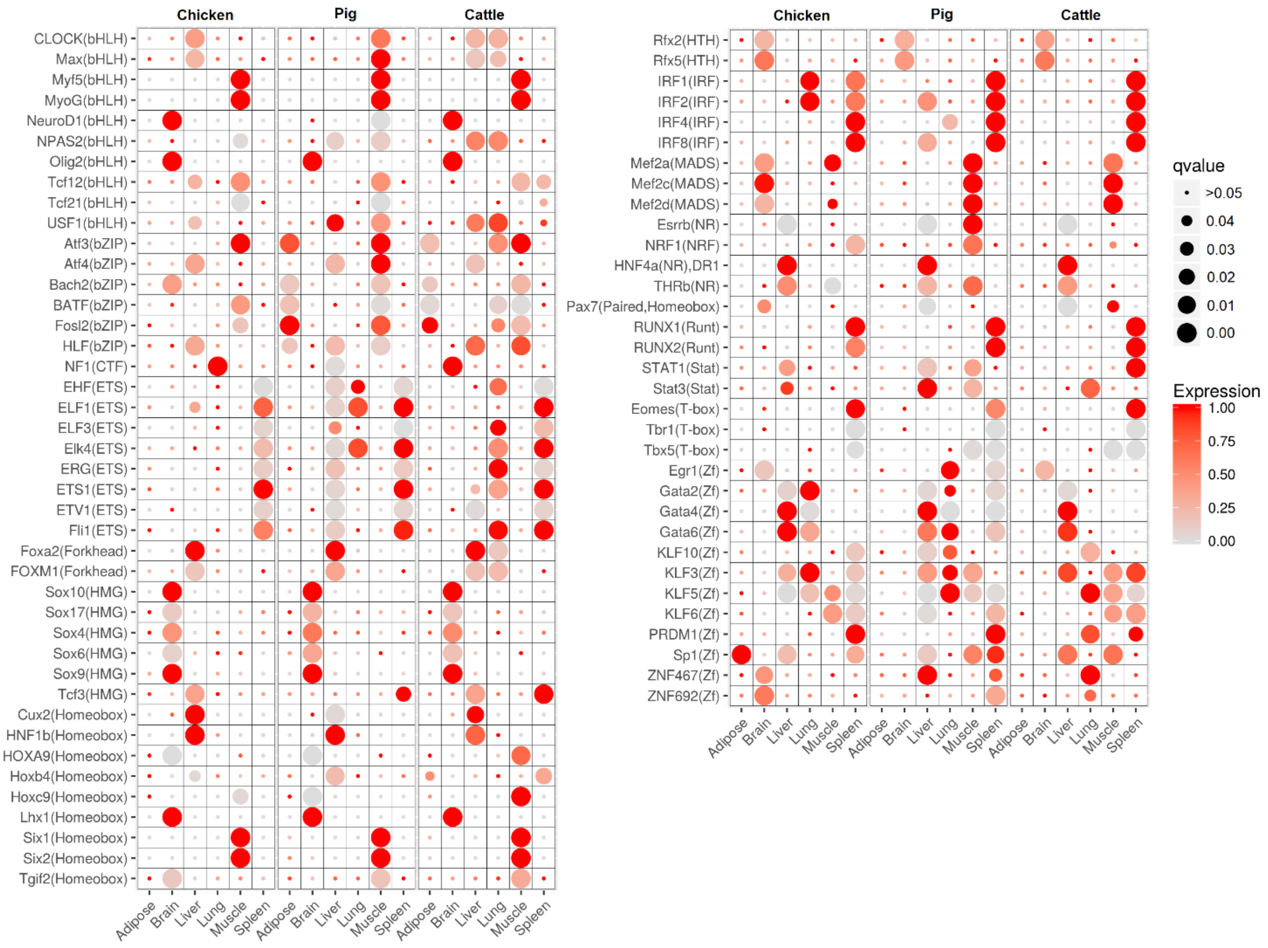

Supplementary Figure 8. **Transcription factors motifs enriched in regulatory elements predicted to target tissue-specific genes.** Transcription factor motifs enriched in at least one tissue in all three species based on footprints lying within regulatory elements predicted to target tissue-specific genes. The size of the circle indicates the statistical significance of motif enrichment, while the color indicates the expression of the corresponding transcription factor gene, normalized to the maximum expression across tissues within each species.

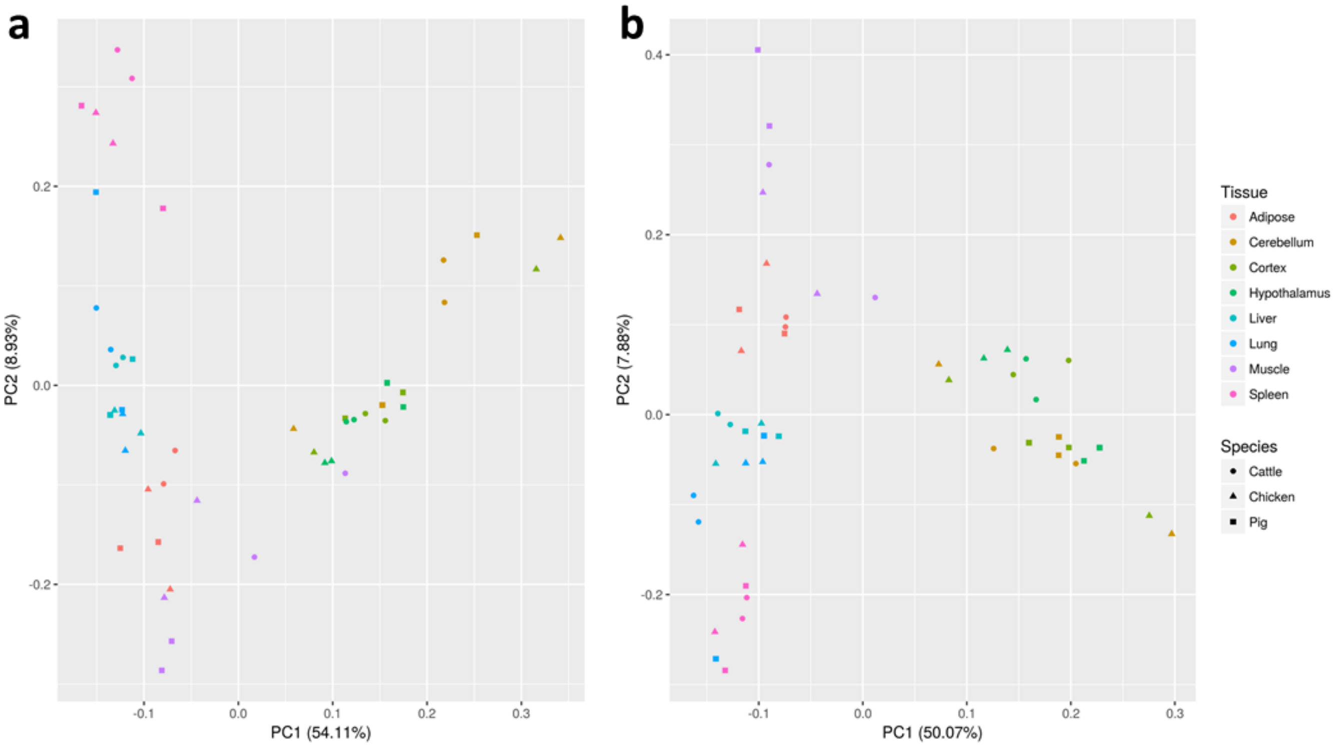

Supplementary Figure 9. **Principal component analysis of TSS proximal and genic regulatory elements targeting orthologs.** Principal component plot of the normalized read depth of H3K27ac of TSS proximal (**a**) and genic (**b**) REs predicted to target genes with one-to-one orthologs across all three species.
