# Supplementary material for: Functional annotations of three domestic animal genomes provide vital resources for comparative and agricultural research
Source: Nat Commun. 2021 Mar 23;12:1821. doi: 10.1038/s41467-021-22100-8 (PMC7988148; doi:10.1038/s41467-021-22100-8)
Supplement: Supplementary file 3 — Description of Additional Supplementary Files [file 41467_2021_22100_MOESM3_ESM.pdf]

Description of Additional Supplementary Files

File Name: Supplementary Data 1
Description: Number of raw, trimmed, aligned, and filtered, and duplicate reads for each sequencing dataset.

File Name: Supplementary Data 2
Description: Quality metrics (NRF, PBC1, PBC2, NSC, RSC, JSD, number of peaks, genome coverage of peaks, and FRiP, see Methods for details of these metrics) for each ChIP-seq dataset.

File Name: Supplementary Data 3
Description: ENCODE data portal accession numbers for human and mouse ENCODE and human Roadmap datasets used in this manuscript.
