# Supplementary material for: Functional annotations of three domestic animal genomes provide vital resources for comparative and agricultural research
Source: Nat Commun. 2021 Mar 23;12:1821. doi: 10.1038/s41467-021-22100-8 (PMC7988148; doi:10.1038/s41467-021-22100-8)
Supplement: Supplementary file 7 — Reporting Summary [file 41467_2021_22100_MOESM7_ESM.pdf]

nature research

Corresponding author(s): Huaijun Zhou

Last updated by author(s): Aug 28, 2020

Reporting Summary

Nature Research wishes to improve the reproducibility of the work that we publish. This form provides structure for consistency and transparency in reporting. For further information on Nature Research policies, see our Editorial Policies and the Editorial Policy Checklist.

Statistics

For all statistical analyses, confirm that the following items are present in the figure legend, table legend, main text, or Methods section.

n/a	Confirmed	
☐	☒	The exact sample size (*n*) for each experimental group/condition, given as a discrete number and unit of measurement
☐	☒	A statement on whether measurements were taken from distinct samples or whether the same sample was measured repeatedly
☐	☒	The statistical test(s) used AND whether they are one- or two-sided<br>*Only common tests should be described solely by name; describe more complex techniques in the Methods section.*
☒	☐	A description of all covariates tested
☐	☒	A description of any assumptions or corrections, such as tests of normality and adjustment for multiple comparisons
☐	☒	A full description of the statistical parameters including central tendency (e.g. means) or other basic estimates (e.g. regression coefficient) AND variation (e.g. standard deviation) or associated estimates of uncertainty (e.g. confidence intervals)
☐	☒	For null hypothesis testing, the test statistic (e.g. F, t, r) with confidence intervals, effect sizes, degrees of freedom and P value noted<br>*Give P values as exact values whenever suitable.*
☒	☐	For Bayesian analysis, information on the choice of priors and Markov chain Monte Carlo settings
☒	☐	For hierarchical and complex designs, identification of the appropriate level for tests and full reporting of outcomes
☐	☒	Estimates of effect sizes (e.g. Cohen's d, Pearson's r), indicating how they were calculated

Our web collection on statistics for biologists contains articles on many of the points above.

Software and code

Policy information about availability of computer code

Data collection	No software was used for data collection
Data analysis	Trim Galore! 0.4.5, STAR 2.5.4a, SAMtools 1.10, htseq-count 0.11.1, edgeR 3.11, BWA 0.7.17, Picard toolkit 2.18.17, SPP, DeepTools 3.3.0, Macs 2.1.1, ChromHMM 1.19, BEDTools 2.27.1, DAVID 6.8, HOMER 4.10, UCSC liftOver.

For manuscripts utilizing custom algorithms or software that are central to the research but not yet described in published literature, software must be made available to editors and reviewers. We strongly encourage code deposition in a community repository (e.g. GitHub). See the Nature Research guidelines for submitting code & software for further information.

Data

Policy information about availability of data

All manuscripts must include a data availability statement. This statement should provide the following information, where applicable:

- Accession codes, unique identifiers, or web links for publicly available datasets
- A list of figures that have associated raw data
- A description of any restrictions on data availability

Raw sequencing data has been deposited in the European Nucleotide Archive (ENA) and is available under accession PRJEB14330. Accession numbers for ENCODE and Roadmap datasets used are given as supplementary information.

Field-specific reporting

Please select the one below that is the best fit for your research. If you are not sure, read the appropriate sections before making your selection.

☒ Life sciences ☐ Behavioural & social sciences ☐ Ecological, evolutionary & environmental sciences

For a reference copy of the document with all sections, see nature.com/documents/nr-reporting-summary-flat.pdf

Life sciences study design

All studies must disclose on these points even when the disclosure is negative.

Sample size	Two biological replicates per tissue were used per species, which was chosen to follow the experimental design of ENCODE Consortium and FAANG Consortium
Data exclusions	Data for only 1 replicate for DNase-seq from chicken hypothalamus was generated because the lab generating the data could not produce a successful library from the other sample. ATAC-seq data from only 1 replicate was used from cattle cerebellum as the sample for the other replicate could not be located.
Replication	Technical replicates were not performed.
Randomization	This is not relevant to our study as we did not separate the individuals into experimental groups.
Blinding	This is not relevant to our study as we did not separate the individuals into experimental groups.

Reporting for specific materials, systems and methods

We require information from authors about some types of materials, experimental systems and methods used in many studies. Here, indicate whether each material, system or method listed is relevant to your study. If you are not sure if a list item applies to your research, read the appropriate section before selecting a response.

Materials & experimental systems

n/a	Involved in the study	
☐	☒	Antibodies
☒	☐	Eukaryotic cell lines
☒	☐	Palaeontology and archaeology
☐	☒	Animals and other organisms
☒	☐	Human research participants
☒	☐	Clinical data
☒	☐	Dual use research of concern

Methods

n/a	Involved in the study	
☐	☒	ChIP-seq
☒	☐	Flow cytometry
☒	☐	MRI-based neuroimaging

Antibodies

Antibodies used	All antibodies used were from Diagenode. For chicken and pig ChIP-seq, catalog #C01010059 lot A17723-0041D, catalog #C15410069 lot A1821D, catalog #C15410174 lot A1723-0041D, catalog #C15410037 lot A1862D, and catalog #15410210 lot A2359-00234P for H3K4me3, H3K27me3, H3K27ac, H3K4me1, and CTCF, respectively. For cattle, the same lots were used for H3K27ac, H3K4me1, and CTCF, while lot A1051D for H3K4me3 and lot A00821D for H3K27me3 were used with the same catalog numbers as for chicken and pig.
Validation	Validation was performed by Diagenode as stated on their website: https://www.diagenode.com/en/categories/antibodies

Animals and other organisms

Policy information about studies involving animals; ARRIVE guidelines recommended for reporting animal research

Laboratory animals	Chickens were 20 week old male F1 crosses of Line 6 and Line 7, pigs were 6 month old castrated male Yorkshires, and cattle were 14 month old intact male Line 1 Herefords.
Wild animals	This study did not involve wild animals
Field-collected samples	This study did not involve samples collected from the field
Ethics oversight	Protocol for Animal Care and Use #18464, approved by the Institutional Animal Care and Use Committee (IACUC), University of California, Davis

Note that full information on the approval of the study protocol must also be provided in the manuscript.

ChIP-seq

Data deposition

- [x] Confirm that both raw and final processed data have been deposited in a public database such as GEO.
- [x] Confirm that you have deposited or provided access to graph files (e.g. BED files) for the called peaks.

Data access links<br>*May remain private before publication.*	Raw data has been deposited at https://www.ncbi.nlm.nih.gov/geo/query/acc.cgi?acc=GSE158430. Processed data under the folder "Processed_Reads" and BED files for the called peaks under the folder "Peak_Calls" is available at http://farm.cse.ucdavis.edu/~ckern/Nature_Communications_2020/.
Files in database submission	A total of 339 fastq files were generated
Genome browser session<br>(e.g. UCSC)	Links to UCSC genome browsers for the 3 species are here: http://farm.cse.ucdavis.edu/~ckern/FAANG/

Methodology

Replicates	2 biological replicates were used per tissue. Replicate agreement was measured by Pearson correlation of read depth in genomic bins by deepTools version 3.3.0 and is included in extended data Figures 2-4.
Sequencing depth	All ChIP-seq data were single-end 50bp reads. A minimum of 20 million uniquely mapped reads was targeted for all marks except H3K27me3 where 40 million uniquely mapped reads was targeted. The average depth per library is reported in Extended Data Table 1, and the sequencing depth of all 240 ChIP-seq libraries can be provided in supplementary information.
Antibodies	All antibodies used were from Diagenode. For chicken and pig ChIP-seq, catalog #C01010059 lot A17723-0041D, catalog #C15410069 lot A1821D, catalog #C15410174 lot A1723-0041D, catalog #C15410037 lot A1862D, and catalog #15410210 lot A2359-00234P for H3K4me3, H3K27me3, H3K27ac, H3K4me1, and CTCF, respectively. For cattle, the same lots were used for H3K27ac, H3K4me1, and CTCF, while lot A1051D for H3K4me3 and lot A00821D for H3K27me3 were used with the same catalog numbers as for chicken and pig.
Peak calling parameters	Macs 2.1.1 was used to call peaks, with a q-value threshold of 0.01 used for H3K4me3, H3K4me1, H3K27ac, and CTCF, while a q-value threshold of 0.05 and the broad peak calling flag was used for H3K27me3.
Data quality	Data quality metrics used are described in the methods of the manuscript, and include the non-redundant fraction, PCR bottleneck coefficients, fraction of reads in peaks, normalized and relative strand correlations, and Jensen-Shannon distance. The average metrics per library are reported in Extended Data Table 1, and a table with the metrics for each individual library can be included in the supplementary information.
Software	Trim-galore 0.4.5 was used to trim reads, then aligned with BWA 0.7.17. Picard tools 2.18.17 was used to mark and filter PCR duplicates. ChromHMM 1.19 was used to predict chromatin states. DeepTools 3.3.0 and BEDTools 2.27.1 were used for a variety of analysis tasks. The data processing pipeline is available at https://github.com/kernco/functional-annotation
